# Role of human milk oligosaccharide metabolizing bacteria in the development of atopic dermatitis/eczema

**DOI:** 10.3389/fped.2023.1090048

**Published:** 2023-03-20

**Authors:** Trisha Rahman, Prioty F. Sarwar, Cassie Potter, Sarah S. Comstock, Vanja Klepac-Ceraj

**Affiliations:** ^1^ Department of Biological Sciences, Wellesley College, Wellesley, MA, United States; ^2^ Department of Food Science & Human Nutrition, Michigan State University, East Lansing, MI, United States

**Keywords:** atopic dermatitis, eczema, human milk oligosaccharides (HMO), infant gut microbiome, bifidobacterium infantis, metagenome, metabolome, HMO metabolizing genes

## Abstract

Despite affecting up to 20% of infants in the United States, there is no cure for atopic dermatitis (AD), also known as eczema. Atopy usually manifests during the first six months of an infant's life and is one predictor of later allergic health problems. A diet of human milk may offer protection against developing atopic dermatitis. One milk component, human milk oligosaccharides (HMOs), plays an important role as a prebiotic in establishing the infant gut microbiome and has immunomodulatory effects on the infant immune system. The purpose of this review is to summarize the available information about bacterial members of the intestinal microbiota capable of metabolizing HMOs, the bacterial genes or metabolic products present in the intestinal tract during early life, and the relationship of these genes and metabolic products to the development of AD/eczema in infants. We find that specific HMO metabolism gene sets and the metabolites produced by HMO metabolizing bacteria may enable the protective role of human milk against the development of atopy because of interactions with the immune system. We also identify areas for additional research to further elucidate the relationship between the human milk metabolizing bacteria and atopy. Detailed metagenomic studies of the infant gut microbiota and its associated metabolomes are essential for characterizing the potential impact of human milk-feeding on the development of atopic dermatitis.

## 1. Introduction

Atopic dermatitis (AD), commonly referred to as eczema, is an inflammatory skin condition that affects up to 20% of infants. This condition is a growing problem in the United States, as higher percentages have been reported in more recent years (1–4). Development of atopy in early life is also one of the predictors of later allergic or other hyperinflammatory health problems, such as asthma, rhinitis or food allergies (5). Symptoms usually begin during the first six months of an infant's life and include itchy and painful skin and sleep disruption (6–8). Not only does this disease decrease quality of life for infants with AD/eczema and their families (9, 10), children with AD/eczema also have more hospital visits and hospitalizations than those without AD/eczema (11). Families also experience substantial financial burden; the annual cost for both adults and children in the United States with AD/eczema was estimated to be $5.3 billion in 2015 (12), with out-of-pocket expenses disproportionately affecting Black Americans (13). Thus, this is a highly prevalent immune condition that negatively impacts infants and their families, where early interventions may prevent later negative health outcomes.

There is a genetic predisposition for AD/eczema, but genetics can only explain a percentage of AD/eczema cases. Mutations in the FLG gene are known to be associated with the development of AD/eczema because the lack of the structural protein filaggrin leads to impairment of the barrier function of the skin. FLG gene mutations, however, explain up to only 50% of AD/eczema cases (14, 15). In addition to genetics, gut microorganisms have been implicated in the development of AD/eczema (16–18). The gut-associated microorganisms affect the development of the immune system by providing substrates and metabolites that induce and recruit a repertoire of innate immune cells and train long-term adaptive immune responses (17). Gut microbiota-mediated stimulation of regulatory immune pathways leads to many changes in the host, such as changes in gene expression that can lead to the recruitment and proliferation of immune cells, changes in intestinal barrier integrity and the release of antimicrobial peptides, to name a few (19–23). Even though the infant gut microbiome is largely transient, the impact of the early-life gut microbiome on the immune system has been shown to have both positive and negative effects on the development of inflammatory diseases during infancy (24).

Human milk shapes the early infant gut microbiota, and in this way, plays key roles in modulating the development of the innate and adaptive mucosal immune systems and regulating gut barrier integrity (25). One important component of human milk is human milk oligosaccharides (HMOs), a group of prebiotic oligosaccharides and the third most abundant component in human milk after lactose and lipids. An HMO is a soluble glycan consisting of five basic units: one acid monosaccharide, one amino sugar and three monosaccharides. The three major categories of HMOs include fucosylated, sialylated and neutral oligosaccharides (26), and the immunomodulatory effects of each type on innate and systemic immunity has been extensively reviewed by Kulinich and Liu (27), and Donovan and Comstock (17), respectively. HMOs cannot be digested by the human infant and, instead, serve as a carbon and energy source for bacteria (28, 29). There are over 200 known structures of HMOs (30). Several genetic and non-genetic factors result in vastly different HMO repertoires in the milk of lactating individuals (31). Thus, the most abundant HMO varies by lactating person and has been reviewed by others (32, 33).

**Figure 1:**
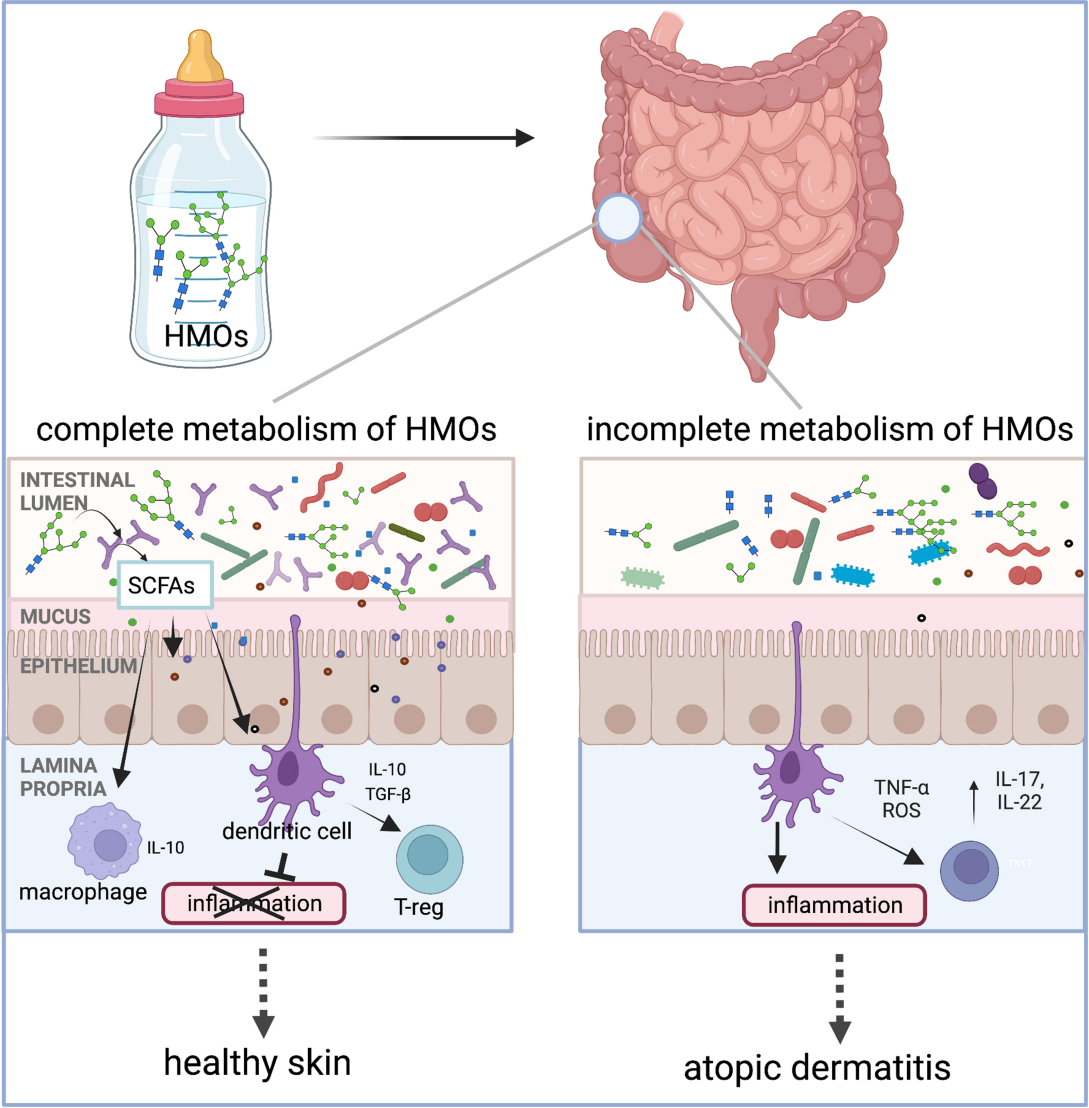
Human milk oligosaccharides (HMOs) are the third most abundant component in human milk after lactose and lipids. Humans cannot digest HMOs. Instead, these carbohydrates are a food source for infant gut microorganisms such as *B. infantis*, *Bacteroides* spp., and others. In the gut, bacteria with HMO metabolizing genes break down complex oligosaccharides into simpler metabolites, such as short-chain fatty acids (SCFAs). These metabolites either signal the immune system through interactions with dendritic cells whose dendrites are sampling the gut lumen or by crossing the epithelial barrier to interact with immune cells in the lamina propria. Interaction between the dendritic cells and specific HMO metabolites results in dendritic cells releasing interleukins, such as IL-10, which are involved in regulating inflammation. We hypothesize that infants with microorganisms that have the complete set of HMO metabolizing genes are protected from the development of AD/eczema and/or experience reduced severity of AD/eczema. Intestinal cells and immune cells have receptors for SCFAs which are not pictured in this simplified schematic but have been reviewed by elsewhere (46). The figure was created in BioRender.com.

There is conflicting evidence regarding the associations between human milk and AD/eczema (Table 1). Several studies and meta-analyses have reported a protective role against AD/eczema when infants are fed a diet of human milk, especially in the first 4 months of life (37–40). Yet several others have found compounding factors that can reduce the effectiveness of a human milk diet in the prevention of AD/eczema, like atopic heredity and the infant's age (40–44). The observed human milk diet-specific protections against AD/eczema potentially stem from differences in the composition of the infant gut microbiota and its ability to completely metabolize HMOs (16). Only a handful of taxa are known to possess HMO metabolizing enzymes, and the prevalence of metabolic machinery capable of metabolizing HMOs is low among microbial taxa residing within the human gastrointestinal tract. Notably, several species from the genus *Bifidobacterium* have the ability to metabolize HMOs, and these organisms have been known to influence neonatal mucosal and systemic immunity (17, 45) via the production of short-chain fatty acids (SCFAs) or direct modulation of the immune system (Figure 1). Hence, differences in types and abundances of HMO metabolizing bacteria may impact host immune development (47, 48). The differential ability of infant intestinal bacteria to metabolize specific HMOs may explain why a diet of human milk protects against AD/eczema in some but not all infants fed human milk.

**Table 1:** Prevalence of AD/eczema in human milk or formula fed infants.

Source	Data Collected	Geography	Age at Sampling	Breastfeeding Duration	Feeding Status: Breast Fed (BF) or Formula Fed (FF)	AD/eczema (%)
Ta et al., 2020 (16)	2009–2020	Singapore	Clinical: 3 weeks; 3, 6, 9, 12, 15, 18, 24 and 36 months Stool: 3 weeks; 3, 6 and 12 months	6 months	Exclusive BF	9.09
					Predominantly BF	24.24
					Partial BF	27.27
					Exclusive FF	29.29
Parazzini et al., 2013 (34)	2007–2009	Italy	Birth, 6 and 12 months	Variable; 5+ months	Exclusive BF	30.1
					BF/ other milks	26.5
					No BF	22.9
Lee et al., 2017 (35)	2007–2012	South Korea	Early life retrospectively of 1 to 3-year old	Variable; up to 11 months	Exclusive BF	36.9
					Mixed Feeding	55.4
					Exclusive FF	7.7
Schoetzau et al., 2008 (36)	1995–98	Germany	1, 4, 8 and 12 months	Variable; up to 6 months	BF	9.5
					FF	14.8

To understand the development of atopy in early life, it is imperative to investigate the links between human milk exposure, variations in HMO metabolic capacity of commensal bacteria and health outcomes such as atopic dermatitis. Atopy, including the development of AD/eczema, is the first step toward a lifetime of disease in many children. Halting the progression of the atopic march (5, 49) is of utmost importance to have positive health outcomes for US infants and children. Herein, we: 1) summarize what is currently known about the microorganisms and genes involved in HMO metabolism, 2) describe patterns of HMO metabolites associated with AD/eczema, and 3) evaluate whether the abundance and diversity of HMO metabolizing genes in breastfed infants can be a predictor of an infant's risk for AD/eczema. We also identify the gaps in our understanding of how microorganisms or their metabolites modulate the infant immune system during atopic disease. To address the topic, we followed the classical methodology for a narrative review (50). A list of keywords such as human milk oligosaccharide metabolizing genes, atopy, atopic march, *Bifidobacterium infantis*, metabolites, short chain fatty acids, immunity, and infant gut microbiome were initially identified. Then, keyword combinations adding the term “eczema” or “atopy” or “atopic dermatitis” were used. We used Google Scholar, PubMed, and Web of Knowledge to search for available literature published in English and indexed either as original articles or reviews. We focused on publications published in the last 5 years, but did not limit our search to a specific time range.

## 2. The connection between human milk oligosaccharides, infant intestinal microbiota and atopy

### 2.1. Microbial composition in the gut of infants fed human milk

Humans and their microbiomes have co-evolved for tens of thousands of years, resulting in a tight partnership between the microorganisms and their host. HMOs have likely played a role in this co-evolutionary relationship as they cannot be digested by infants but instead support the colonization and growth of beneficial microbes in the infant gut (51). In fact, longitudinal sampling of fecal HMOs matched with the infant gut microbiota shows an inverse relationship between the levels of fecal HMOs and the abundance of HMO metabolizing bacteria (52). Therefore, it is not surprising that the infant gut microbial community composition differs in infants fed exclusively human milk compared to those fed no human milk or mixed diets, especially early in life (53–57). Early colonization of the infant gut is variable between individual infants, but human milk fed infants have higher abundances of bifidobacterial species compared to formula fed infants (47, 58). *Bifidobacterium longum* subsp. *infantis*, (*B. infantis*), one of a few microorganisms capable of metabolizing HMOs and one of the pioneer colonizers of the infant gut in breastfed infants, generates metabolites that protect against inflammation, such as HMO-derived SCFAs and aromatic lactic acids (59, 60). Decreased levels of *Bifidobacterium* spp. in the infant gut have been associated with increased incidence of obesity, allergies, and diabetes (61–63). In addition to *B. infantis*, only a few microbes from the *Bifidobacterium*, *Lactobacillus*, *Enterococcus* and *Bacteroides* genera have been shown to encode a substantial number of genes to either partially or fully metabolize HMOs (47, 64–69). These HMO-utilizing bacteria are also routinely detected in the fecal microbiota of neonates (66, 67, 70, 71). Several of these early colonizers of the gut of human milk fed infants affect human immune system development and function (72, 73).

### 2.2. Changing prevalence of *B. infantis* and other HMO metabolizing microorganisms in healthy, human-milk fed infants

Some surveys state that the microbiomes of healthy, human milk fed infants in countries where breastfeeding is widespread are dominated by *Bifidobacterium* at abundances that often exceed 60% (74–77). On the other hand, a generational loss of *Bifidobacterium* can be observed in regions like the US and Europe where there is a high prevalence of formula feeding and disruption of the fecal-oral transfer of maternal gut bacteria to infants due to higher incidence of cesarean births and perinatal antibiotic use (78). It is unclear whether the purported disappearance of specific *Bifidobacterium* taxa from the infant gut coincides with an overall decline of HMO metabolizing microorganisms, but it is clear that functional differences in the gut microbiota can be attributed to lifestyle, with the presence of more complex HMO degradation machinery associated with non-industrialized infants (79).

However, some studies suggest a low relative abundance of *Bifidobacterium* in infants, at levels of ∼30% or lower, regardless of geography, industrialization and breastfeeding status (80, 81). Reduced abundance and prevalence of *Bifidobacterium* may be due to the presence of other HMO metabolizing microbes from the genera *Lactobacillus* or *Bacteroides* that compete for the same resources as members of the genus *Bifidobacterium* (68, 71). Alternatively, discrepancies in reports may also be tied to a lack of sensitivity in the detection of *Bifidobacterium* taxa (82–84). For example, *B. infantis* is challenging to detect and identify by PCR-based methods using the 16S rRNA gene as its 16S rRNA gene is almost identical to that of the closely related *B. longum* and *B. suis* strains (85). Both the sequencing technique (52) and the use of different 16S rRNA gene primer pairs (83) lead to a varying degree of sensitivity in detecting and identifying *B. infantis*. More recently, studies using metagenomic approaches have indeed observed a decline of *B. infantis* in industrialized populations as compared to non-industrialized populations (79). Given a true decline in HMO metabolizing capacity in the infant gut, restoring these missing microorganisms could prevent pediatric diseases such as islet autoimmunity, type I diabetes, and intestinal disorders (86, 87). Despite these reports that suggest the importance of HMO metabolizing bacteria in pediatric diseases, our understanding of the prevalence of HMO metabolizing bacteria in present infant populations is limited. Furthermore, the association of these bacteria with specific intensities of human milk exposure has only been evaluated to a limited extent (29).

### 2.3. HMO metabolizing genes

Only a subset of bacteria possess the enzymatic machinery that is required to break down, metabolize, and grow on HMOs (Table 2) (86). These microbes are highly specialized such that they typically disappear when HMOs are removed from the diet (53, 108). *B. infantis* has the majority of HMO metabolizing genes that have been discovered and characterized to date (60, 109), and these genes can be classified into three categories: extracellular glycosidases, transporters, and intracellular enzymes. Bacteria use multiple combinations of these HMO metabolizing gene types in two different HMO metabolizing strategies (110). One strategy involves the hydrolysis of the HMO extracellularly into mono- and disaccharides using cell wall-anchored secretory glycosidases, followed by saccharide transport into the cells (111). The second strategy is to directly import HMOs with ≥3 sugar subunits into the cells via ATP-binding cassette (ABC) transporters followed by intracellular HMO hydrolysis by exoglycosidases (112). HMO metabolizing gene cassettes also have been found in some species of *Bacteroides* (66). Some members of the genus *Bifidobacterium* or *Lactobacillus* possess incomplete metabolic machinery and may rely on other strains for completion of HMO digestion or may cross-feed on digested or partially digested metabolites from other bacteria (65).

**Table 2:** Select bacterial species and their association with AD/eczema and HMO metabolizing capacity.

Genus	Species	Present in infant gut	Association with AD/eczema	HMO metabolizing capability	Notes
*Bifidobacterium*	*longum* subsp. *infantis*	Yes	Inverse association (88); No association (89)	Yes (85)	5 known gene clusters; metabolizes several small mass HMOs
	*longum* subsp. *longum*	Yes	Inverse association (88); No association (89)	No (90)	Ferments plant oligosaccharides and their constituent pentose sugars
	*breve*	Yes	Inversely associated in the first 12 months (89)	Yes (91, 92)	Grows on small mass HMOs; HMO substrate from mothers milk
	*bifidum*	Yes	Inverse association (88)	Yes (91–93)	Grows on 3′-sialyllactose, LNBs; HMOs with type 1 chain; HMO substrate from mothers milk
	*catenulatum*	Yes	Positive association at 3 months (89)	Potential (94)	subsp. *kashiwanohense* encode HMO metabolizing genes
	*animalis* subsp. *animalis*	No (95)	N/A*	Yes (96)	Some strains can grow on lacto-N-biose I
	*animalis* subsp. *lactis*	Yes	No association (89)	Weak (97)	Grows weakly in 3-fucosyllactose
*Bacteroides*	*fragilis*	Yes	Inverse association (16, 88)	Yes (98)	Grows on 2% HMO defined medium
*Lactobacillus*	*casei*	Yes (99)	N/A	Yes (100)	Specifically metabolizes N-acetyllactosamine (LacNAc)
	*acidophilus*	Yes	Inverse association as part of a probiotic mix (101)	Yes (67, 98)	Releases mono- and disaccharides from defined HMOs; grows on chemical growth medium of 2% HMOs
*Escherichia*	*coli*	Yes	Positive association (16)	Weak (98)	One strain shows weak growth on chemical growth medium of 2% HMOs
*Staphylococcus*	*aureus*	Yes	Positive association (102)	No (103)	HMOs enhances growth but species does not metabolize HMOs
*Klebsiella*	*pneumoniae*	Yes	Positive association (16)	Some (104)	Does not grow on 2′-fucosyllactose or 6’-sialyllactose; grows on mono- and disaccharide components of HMOs
*Faecalibacterium*	*prausnitzii*	Yes	Positive association (88)	Weak (90)	Weakly grows on tested HMOs
*Ruminococcus*	*gnavus*	Yes	Positive association (88)	Potential (105)	Expresses HMO related degradation enzymes
*Akkermansia*	*muciniphila*	Yes	Positive association (88)	Yes (106, 107)	Expression of key glycan degrading enzymes when grown on human milk; strain dependant

^*^ N/A used when information in the literature was not found. (number) is the citation.

*B. infantis* is unique for its presence of five gene clusters that encode enzymes that bind, cleave and transport HMOs (109, 113), but little is known about the specific targets of each gene cluster. We have recently explored publicly available genomes within the *B. longum* group: *B. longum infantis*, *B. longum longum* and *B longum suis*. Using average nucleotide identity (ANI) which is a measure of nucleotide-level genomic similarity between the coding regions of two genomes, we were able to show that *B. infantis* clustered separately from other relatives of the *B. longum* group, and individual *B. infantis* genomes contained all or almost all HMO metabolizing genes associated with the five clusters. *B. longum* and *B. suis* were missing these genes, although several *B. longum* genomes contained a complete set of genes from cluster four but lacked genes from the other clusters (85). Notably, the metabolites resulting from HMO degradation processes can feed other members of the gut bacterial community or signal to the host via immune cells, enterocytes or other cells in the gastrointestinal tract (17, 65, 114).

Microbial species are formed by acquisition and loss of functional traits by horizontal gene transfer (HGT), gene loss, duplication, and selection. These events are important in the evolution of microbial genomes (115). Thus, even in closely related microorganisms belonging to the same species or species group, gene content can vary significantly (116, 117). The *B. longum* group comprising of three subspecies is a good example of such variation. Additionally, the gene content can also vary significantly across metagenomes (47, 69). In fact, two different microorganisms can use different metabolic pathways to perform the same function. For example, *Bacteroides* and *Bifidobacterium* are both present in the infant gut and can digest HMOs. However, *Bacteroides* use the set of genes they typically use to metabolize host mucus glycans to metabolize HMOs (66) and these genes do not overlap with the HMO metabolism genes encoded by *B. infantis* (66). Moreover, even within *Bacteroides* spp., the genes for HMO utilization are not conserved, and *B. fragilis* and *B. thetaiotaomicron* use different sets of genes to metabolize identical HMO (66, 98). Low conservation of HMO metabolizing gene sets across genera and even within genera illustrates an important challenge in identifying other HMO utilizing bacteria. It is important to note that the lack of known HMO utilization genes in a species is not indicative of a lack of HMO metabolizing capacity as the species may use an entirely different set of genes for HMO metabolism. Thus, analyses of the presence and abundance of specific genes rather than cataloging specific bacterial taxa will be required to fully understand any associations between HMOs and the development of AD/eczema in infants.

### 2.4. HMO metabolizing bacteria and their metabolites: impact on the developing immune system and atopy

HMOs are known to influence neonatal mucosal and systemic immunity directly and indirectly (17, 45, 51). For example, HMOs increase intestinal cell maturation and barrier function (118, 119), and directly interact with immune cells to influence proliferation and cytokine production (17). Some HMOs, particularly those carrying fucosyl groups, are associated with a Th2 promoting anti-inflammatory response (27). The loss of anti-inflammatory signals can indicate a shift in the homeostatic environment of the GI tract to favor chronic inflammation (120). Furthermore, differences in types and abundances of bacteria capable of metabolizing HMOs may also play a role in host immune development (47, 48), but understanding how these microorganisms or their metabolites modulate the immune system is still in its infancy. Both human milk feeding (17, 51) and the presence of *Bifidobacterium* spp. (121, 122), have been previously reported to protect children from AD/eczema. However, few studies to date have conducted a detailed analysis of (1) HMO metabolizing genes or (2) HMO metabolites and their patterns of association with AD/eczema.

Once in the gut, the HMOs are metabolized by gut microbiota, most commonly by a member from the genus *Bifidobacterium*, into simpler carbohydrates and SCFAs (123). SCFAs interact with the immune system in various beneficial ways. For instance, they assist with maintaining intestinal epithelial barrier integrity, increase key gene expression and modulate dendritic cells and T cells (114). Butyrate, propionate, and acetate are the most notable SCFAs that influence immune health, with isobutyrate, isovalerate, valeric acid, and n-butyric acid also being present in infant guts (124). The presence of *Bifidobacterium* species in the infant gut, associated predominantly with breastfeeding, leads to the production of aromatic lactic acids from HMOs, specifically indolelactic acid, phenyllactic acid and 4-hydroxyphenyllactic acid. These *Bifidobacterium* species also produce amino acids, specifically tryptophan, phenylalanine and tyrosine (123, 125, 126). In particular, it has been shown with both *in vitro* and *ex vivo* methods that fecal concentrations of *Bifidobacterium*-derived indolelactic acid can activate the aryl hydrocarbon receptor important for controlling intestinal homeostasis and immune responses (125). Furthermore, two metabolites of tryptophan, an amino acid product of HMO degradation nicotinamide and 3-hydrokynurenine, are associated with increased atopy. But the association of increased levels of nicotinamide in maternal plasma and the development of infant AD/eczema conflict in the literature (127, 128).

## 3. Evidence for the role of HMO metabolizing microorganisms and their metabolites in atopic dermatitis (AD)/eczema

### 3.1. Patterns of HMO metabolizing bacteria/genes with AD/eczema

Several studies have reported associations of bacterial taxa with AD/eczema as summarized in Table 2. At the phylum level, reduced diversity of *Bacteroidetes* has been observed in one-month old infants with AD/eczema, while reduced diversity of *Proteobacteria* has been associated with AD/eczema in 12 month old infants (129). Although not known to metabolize HMOs, several bacterial genera have been found in greater abundance in infants with AD/eczema and these include *Streptococcus, Escherichia, Shigella, Veillonella, Faecalibacterium, Lachnospiraceae incertae sedis* and *Clostridium XlVa* (88, 130)*.* In terms of specific species, *Faecalibacterium prausnitzii*, *Ruminococcus gnavus, Bacteroides clarus, Bacteroides plebeius, Parabacteroides merdae, Prevotella buccae, Gemmiger formicillis, Akkermansia muciniphila, Hungatella hathewayi* and several members of the families *Bacteroidaceae, Clostridiaceae,* and *Enterobacteriaceae,* have been found in greater abundance in infants with AD/eczema (88, 131, 132). These associations can provide the first step in identifying candidates for further study of HMO metabolizing activity since our understanding of HMO metabolism and the identity of HMO metabolizing genes is still relatively nascent.

In contrast, *Bifidobacterium* and *Bacteroides* genera are present in higher abundances in infants without AD/eczema. Specifically, *Bifidobacterium breve* is associated with a decreased risk of AD/eczema development especially in the first 12 months of life (88, 89, 129). A few *Bifidobacterium* species, such as *B. adolescentis, B. lactis* and *B. longum* subsp. *longum* were found in comparable abundance in infants with and without AD/eczema while *B. catenulatum* was associated with a higher risk of developing AD/eczema (89). None of these bifidobacteria are known to metabolize HMOs completely, and at best, partially metabolize HMOs. There are also non-bifidobacterial species found in lower abundance in infants with AD/eczema, like *Bacteroides fragilis* and *Streptococcus salivarius*, with *B. fragilis* able to partially metabolize HMOs (16, 88). A few members of the genera *Lactobacillus* have also been found to have the ability to metabolize HMOs, namely *L. casei* and *L. acidophilus.* Even though studies of microbiomes associated with AD/eczema do not identify any associations between *Lactobacillus* in the infant gut and the development of AD/eczema, some prenatal or neonatal probiotic interventions with *Lactobacillus* species, like *L. acidophilus* or *L. rhamnosus*, find reduced eczema cases in treatment groups, although with varying levels of efficacy (101, 133–136).

### 3.2. Patterns of HMO metabolites with atopic dermatitis/eczema

The association between AD/eczema and HMO metabolites is still poorly described and is complicated by the variation in the amounts of HMO derived SCFAs at different stages of infancy. Wopereis et al. (137) noted that stool samples of infants with AD/eczema have decreased amounts of lactate and increased propionate and butyrate at 12 weeks. However, the opposite was observed with increased lactate and decreased propionate and butyrate at 26 weeks, with another decrease in butyrate levels by 12 months of age. Ta et al. (16) noticed a similar pattern of low butyrate and propionate levels in the stools of infants with AD/eczema, with AD/eczema infants in their cohort exhibiting consistently low butyrate and propionate levels even at 12 weeks compared to infants with healthy skin. Increasing severity of AD/eczema correlated with a decrease in butyrate-producing bacteria, suggesting there is less butyrate in the gastrointestinal tracts of infants with severe AD/eczema compared to those with milder or no AD/eczema (138). However, other studies have observed similar levels of butyrate in stool samples from infants with or without AD/eczema (130, 139). Another metabolite that is decreased in infants with AD/eczema is valerate/valeric acid, and infants with higher valerate are also protected from later development of AD/eczema (130, 139, 140). Butyrate and valerate were negatively correlated with the genus *Streptococcus* and were decreased in infants with AD/eczema (130). Several studies investigated the fecal metabolites from human milk fed infants (Table 3). In these studies, HMO metabolites in stool easily differentiated formula fed from human milk fed infants. To some extent, the presence of specific SCFAs in feces could differentiate human milk fed from formula fed infants. Dotz et al. (143) found that their cohort of exclusively human milk fed infants could be clustered into three groups characterized by their fecal HMO metabolites as compared to the HMO profiles in mother's milk. These three profiles include (1) infant fecal HMOs with similar or higher complexity than the HMOs in mother's milk, (2) reduced HMO diversity and intensity but increased number and intensity of non-HMOs than mother's milk, or (3) decreased relative intensities of fucosylated HMOs. The acetylated neutral HMOs identified by Dotz et al. (143) in infant and maternal urine have been determined by others to be important in human immunology (150), making this metabolite a candidate metabolite important for the development of atopy and of specific interest to the present review. Similarly, a relationship between *Bifidobacterium* spp., which encodes many HMO metabolizing genes, is found in higher abundance in infants without AD/eczema (60, 89), and fewer fecal HMO metabolites have been observed in infants lacking *B. infantis* (151). Other studies have reported that infants consuming lacto-N-fucopentaose-rich human milk (152) and infants whose mothers have the gene responsible for making 2'fucosyllactose (FUT2) (153) are protected from atopic diseases.

**Table 3:** Stool metabolites as they relate to infant diet and/or AD/eczema.

Study	Diets	Methodology	Metabolites
Chow et al., 2014 (141)	Exclusive human milk; exclusive formula	Untargeted (>250 metabolites)	Human milk fed: more HMOs & their metabolites; fewer protein fermentation metabolites
Martin et al., 2014 (142)	Exclusive human milk; exclusive formula	Untargeted	Similar metabolite profiles between 3 and 6 months: higher propionate, butyrate, acetate, 5-amino-valeratein samples from human milk fed Formula Fed: higher free amino acids (phenylalanine, tyrosine, leucine, and isoleucine)
Dotz et al., 2015 (143)	Exclusive human milk	Targeted: HMO and HMO metabolites	Infants separated into 3 groups of HMO metabolites: 1) more diverse HMOs 2) less diverse HMOs and more non-HMOs 3) less fucosylated HMOs than mother's milk
Kim, H. K., et al, 2015 (144)	Human milk, mixed, formula	Untargeted	Low levels of SCFAs (acetate, propionate, butyrate) in infant group with eczema
Martin et al., 2016 (145)	Exclusive human milk; exclusive formula; mixed-fed	Targeted: SCFAs	Human milk fed: higher proportion of lactate; lower proportion of butyrate Solid food: lower proportion of succinate, lactate; more butyrate
Wopereis et al., 2017 (137)	Exclusive human milk; exclusive formula	Targeted	Unique temporal changes of SCFAs (acetic, propionic, n-butyric, isobutyric, and n-valeric acid) and lactate between eczema and non eczema infants between 12 weeks and 12 months
Bazanella et al., 2017 (146)	Exclusive human milk; exclusive formula	Untargeted and Targeted: SCFA, HMO	Human milk fed differed from formula fed in sterol lipids, glycerophospholipids, fatty acids Human milk fed: lower proportions of propionate, butyrate, valerate and isovalerate; higher proportions of pyruvate and lactate
Bridgman et al., 2017 (147)	Exclusive human milk; exclusive formula; mixed-fed	Untargeted	Human milk fed: higher proportion of acetate than formula fed
Roduit, C., et al. 2019 (148)	Human milk for differing amounts of time (0, <0–2 months, 2–6 months, etc.)	Untargeted	Lower butyrate and propionate levels associated with atopy
Brink et al., 2020 (149)	Human milk; dairy or soy formula	Untargeted	Human milk fed: higher proportion of butyric acid, d-sphingosine, kynurenic acid, indole-3-lactic acid, indole-3-acetic acid, betaine, dopamine
Ta et al., 2020 (16)	Exclusive; predominant; partial human milk; exclusive formula	Untargeted	Lower butyrate and propionate levels in infants with eczema
Laursen, et al., 2021 (125)	Partially human milk, weaned	Untargeted	Both feed types: contains aromatic lactic acids [indolelactic acid, phenyllactic acid and 4-hydroxyphenyllactic acid] converted by *Bifidobacterium* species
Komatsu et al., 2021 (18)	Exclusive human milk	Untargeted	HMOs detected in infant feces up to 4 months: 2′-fucosyllactose, 3- fucosyllactose, 3′-sialyllactose and 6′- sialyllactose

### 3.3. Importance of cross-feeding as it relates to HMO metabolizing microbes

There is some evidence that bacteria which use an extracellular glycosidase-dependent strategy share HMOs and metabolites with other microbes, whereas those bacteria which use an oligosaccharide transporter-dependent method for metabolizing HMOs may inhibit other bacteria from using the HMOs or the resulting HMO metabolites (64). Further, when *B. bifidum*, *B. breve*, and *B. infantis* were present in combination and grown on HMOs, growth was improved compared to that achieved when grown as single strains. Other recent work has demonstrated that HMO metabolizing *Bifidobacterium* spp. can also provide substrates for the growth of non-HMO metabolizing microbes (91). We hypothesize that the diversity of HMO metabolizing genes present in the infant gut microbiome plays an important role in the ability of human milk feeding to reduce the risk for AD/eczema. Thus, approaches which address the full complement of such genes are needed to advance the field.

### 3.4. Role of HMO and HMO derived metabolites in preventing inflammation

In addition to their indirect role in immune development by shaping the infant gut microbiome, HMOs also have direct effects on intestinal maturation and immune development. HMOs can suppress cell cycle progression in intestinal cells to either induce differentiation or cause apoptosis (154). HMOs can also protect the gut against colonization by pathogenic bacteria due to their structural similarities to cell surface glycoconjugates that are recognized by microbes (45). HMOs further interfere with the adhesion of pathogenic bacteria to the intestinal epithelium by modifying the extracellular glycosylation of epithelial cells (154).

HMOs interact with the host's immune system through binding to lectin and TLR receptors. The presence of lectin receptors on a variety of immune cells like macrophages, dendritic cells, neutrophils, eosinophils, monocytes and natural killer (NK) cells (155) and also the detection of HMOs in the plasma of infants consuming human milk diets suggest a direct interaction between HMOs and immune cells both in the GI tract and in the blood (27). Even though direct evidence of the immunomodulatory effect of HMOs during AD/eczema development is sparse, the anti-inflammatory properties of HMOs and their ability to affect tolerogenic factors important for the prevention of allergic diseases have been described in several *in vitro* and *in vivo* studies. In particular, fucosylated HMOs have been associated with a Th2-promoting anti-inflammatory response (27, 154), while sialylated HMOs assist in lymphocyte maturation and maintain the balance between cytokine production related to Th1/Th2 type T-helper responses (155) and also inhibit TLR4/NLRP3 inflammosome pathways (156).

At the intersection of the immunomodulatory effects of HMOs and HMO utilizing bacteria is the effect of HMO derived SCFAs on the infant immune system. While the direct relation between SCFAs and AD/eczema development is still unclear, we can hypothesize that the immunomodulatory effects of metabolites representing unique AD/eczema metabolic signatures may play a role in the development of eczema. SCFAs, specifically propionate and butyrate which are often found in lower levels in individuals with eczema, have been shown to protect individuals from food allergies. The protective effect of SCFAs is thought to be via the development of a regulatory T cell network in the GI tract (157, 158). Butyrate, in particular, can regulate several immunomodulatory pathways by inhibiting histone deacetylases and altering epigenomic signatures (159). Epigenetic modulation is one of the ways that has been identified for microbes to have a long lasting effect on immunity from early childhood exposures (19, 20).

## 4. Discussion

This review addresses fundamental gaps in our understanding of the ecology and evolution of HMO metabolizing microorganisms as well as their association with the prevalent disease of infancy and early childhood, AD/eczema. Though current evidence suggests a protective effect of human milk, enough conflicting studies exist to question the association. Herein, we presented evidence for diversity of HMOs, microbes, and metabolites among even exclusively breastfed infants and suggested that specific repertoires of microbial genes and gene products may be required in order for human milk to protect infants from AD/eczema. In the discussion, we propose possible future research which could elucidate the role of these specific genes and metabolites in human health and disease.

Up to one in five infants in the US experiences AD/eczema. While genetics, such as modifications in the filaggrin gene, can explain a fraction of eczema cases, other factors like the immunomodulatory effect of gut microbes also can play a part in AD/eczema development (16–18). Furthermore, oligosaccharides in human milk serve as a prebiotic to shape the infant gut microbiota, promote growth of commensals and confer immunomodulatory effects to the infant immune system both directly and indirectly. Future research should take an innovative approach to defining factors associated with the developing gut microbiota that could protect from AD/eczema. Although diet is likely an important contributor to this disease, the composition of the microbiota and its ability to digest a specific component of the diet is potentially just as important in protecting against AD/eczema. In fact, the ability of the intestinal microbiota to metabolize distinct sets of HMOs (Table 2) may explain why human milk feeding protects against AD/eczema in some but not all infants fed human milk. Within the context of the host, both the microbial genetic potential for HMO metabolism as well as the resulting metabolites should be examined. While human milk exposure has been suggested to be protective from AD/eczema in observational studies, the evidence presented in this review suggests that specific HMO metabolizing phenotypes may underlie the inconsistent effect of human milk on the prevention of atopy. The HMO metabolites are likely important as these metabolites are known to protect against pathogens and to interact with the infant's developing immune system (51, 160).

In addition to HMOs, there are other immunomodulatory components in human milk, and these can add complexity to how we interpret any observed immunoprotective effects of HMOs. Human milk contains immunoglobulins like secretory IgA and IgG, enzymes such as lysozymes and other factors like lactoferrin and these molecules provide protection from pathogens. Maternal antibodies in the milk can also interact with infant T and B cell programming to influence the adaptive immune response in infants (161). Heterogeneity in the composition of these other human milk components, varying between individuals and even pregnancies in the same individual, may also have a role in the development of atopy.

Measuring HMOs and the by-products of HMO metabolism in infant fecal samples is critical in order to understand their link to AD/eczema. However, assessing the metabolite content of the fecal samples has challenges and limitations. SCFAs are volatile. Their concentrations may be affected by fecal water content, and SCFAs are also readily adsorbed by the infant upon production (162, 163). This means that fecal SCFA concentrations might not reflect the true SCFA production resulting from HMO metabolism in the gut. Despite these challenges, understanding the metabolic signatures in the infant gut provides important insight into the factors affecting the development of AD/eczema.

Specifically, future work should (1) identify the diversity and abundance of HMO metabolizing genes in human atopic infants, (2) link HMO metabolizing genes to specific microbes, (3) evaluate whether there are geographically specific HMO metabolizing repertoires, (4) determine if specific HMO metabolizing genes are associated with an increased or decreased risk of AD/eczema, (5) determine if specific HMO metabolites are associated with the risk of developing AD/eczema. Such studies must control for a variety of covariates including the infant's non-milk diet, mode of delivery, antibiotic use, maternal pre-pregnancy body mass index, as well as additional medical and behavioral outcomes. Those undertaking such research should have combined expertise in microbial ecology, bacterial genomics, bioinformatics, immunology, atopy and allergy, HMOs, and human milk. Detailed genomic, metagenomic and metabolomic studies of the infant gut microbiota, especially during the human milk-feeding stage, are essential for elucidating the impact of early exposures on health outcomes and development of atopy.

## 5. Outlook/conclusion

Host-associated microbes are important determinants of health. Through their interactions with dietary intake or other environmental exposures, these microbes can exacerbate or protect their host from negative health outcomes. As demonstrated in this literature review, human milk may protect against AD/eczema. However, it is the specific genes of, and thus metabolites produced by, microbes consuming HMOs that are likely the most important determinants of the protective role for human milk. Future work must elucidate the diversity of bacterial metabolism and bacterial metabolites associated with AD/eczema in human infants. Through such work, we can gain an understanding of the mechanisms by which microbes and microbial metabolites modulate the developing immune system. Then we can begin identifying metabolic components and metabolites involved in protection against AD/eczema. Ultimately, this will enable the development of biotherapeutics to upregulate or block identified microbial pathways.
